# Biosynthetic Pathway and Metabolic Engineering of Succinic Acid

**DOI:** 10.3389/fbioe.2022.843887

**Published:** 2022-03-08

**Authors:** Xiutao Liu, Guang Zhao, Shengjie Sun, Chuanle Fan, Xinjun Feng, Peng Xiong

**Affiliations:** ^1^ School of Life Sciences and Medicine, Shandong University of Technology, Zibo, China; ^2^ State Key Lab of Microbial Technology, Shandong University, Qingdao, China; ^3^ CAS Key Laboratory of Biobased Materials, Qingdao Institute of Bioenergy and Bioprocess Technology, Chinese Academy of Sciences, Qingdao, China; ^4^ School of Chemical Engineering, University of Chinese Academy of Sciences, Beijing, China

**Keywords:** succinic acid, biosynthesis pathways, metabolic engineering, CO2 fixation, NADH/NAD + ratio

## Abstract

Succinic acid, a dicarboxylic acid produced as an intermediate of the tricarboxylic acid (TCA) cycle, is one of the most important platform chemicals for the production of various high value-added derivatives. As traditional chemical synthesis processes suffer from nonrenewable resources and environment pollution, succinic acid biosynthesis has drawn increasing attention as a viable, more environmentally friendly alternative. To date, several metabolic engineering approaches have been utilized for constructing and optimizing succinic acid cell factories. In this review, different succinic acid biosynthesis pathways are summarized, with a focus on the key enzymes and metabolic engineering approaches, which mainly include redirecting carbon flux, balancing NADH/NAD^+^ ratios, and optimizing CO_2_ supplementation. Finally, future perspectives on the microbial production of succinic acid are discussed.

## 1 Introduction

Succinic acid is a C4-dicarboxylic acid produced as a key intermediate of the tricarboxylic acid (TCA) cycle. As a widely investigated high-value chemical, it has numerous applications in the fields of agriculture, green solvents, pharmaceuticals, and biodegradable plastics. It can also be chemically converted into a variety of important industrial chemicals such as 1,4-butanediol, butadiene, and tetrahydrofuran. Succinic acid has been identified as one of the 12 value-added bio-based platform chemicals by the United States Department of Energy (DOE) (Werpy et al., 2004). The market potential of succinic acid and its direct derivatives is estimated to be as high as 245,000 tons per year, while the market size of succinic acid-based polymers is expected to be 25 million tons per year (Bozell and Petersen, 2010). In traditional chemical synthesis methods, maleic anhydride from petrochemical feedstocks serves as the key substrate for succinic acid, and Ni or Pd-based catalysts have been employed for the hydrogenation of maleic anhydride to succinic acid (Zhang et al., 2020). Although the conversion rate is high, many problems still exist, such as the complex operations required for synthesis, the high-energy consumption, and the harsh reaction conditions (Louasté and Eloutassi, 2020; Zhang et al., 2020). Therefore, much attention has focused on succinic acid biosynthesis, and to date, some efficient succinic acid bio-producers have been successfully applied for industrial purposes (Willke and Vorlop, 2004; Kequan et al., 2008). Compared with chemical processing, the raw materials of biosynthesis are more widely availableand lower in cost. Crude glycerol (Gao et al., 2016), food processing waste (Li et al., 2019), corn fiber (Chen et al., 2011), cassava root (Thuy et al., 2017), lignocellulose hydrolyzate (Zhang et al., 2020), maltose syrup, and other renewable biomass resources are potential sources of the raw materials for these reactions. Significantly, CO_2_ fixation and utilization processes are an integral part of succinic acid biosynthesis in almost all pathways (Table 1). Theoretically speaking, the biological production of 1 kg of succinic acid can fix at least 0.37 kg CO_2_, which presents an environmental benefit to the use of succinic acid biosynthesis upstream of the production of other chemicals (Wang et al., 2011).

**TABLE 1:** Comparison of different succinic acid biosynthetic pathways.

Pathways	rTCA (PPC)	rTCA (PCK)	rTCA (PYC)	rTCA (MAE)	oTCA pathway	GAC pathway	3HP pathway
Precursors	PEP	PEP	PEP	PEP	Acetyl-CoA	Acetyl-CoA	Acetyl-CoA
	CO_2_	CO_2_	CO_2_	CO_2_	OAA	OAA	CO_2_
Reaction steps	4	4	5	4	5	3	6
ATP/SA (mol/mol)	0	+1	0	+1	+1	0	−2
NADH/SA (mol/mol)	−2	−2	−2	−2	+2	0	−3
CO_2_/SA (mol/mol)	−1	−1	−1	−1	+2	0	−2
CO_2_-fixing enzymes	PPC	PCK	PYC	MAE	NE	NE	ACC, PCC

SA: succinic acid.

NE: non-existent.

Abbreviationr: TCA, the reductive branch of the TCA, cycle; oTCA, the oxidative branch pathway of the TCA, cycle; GAC, the glyoxylate shunt pathway.

(−) means to consume (+) means to produce.

Many anaerobic and facultative anaerobic microbes produce succinic acid as their fermentation end product. These succinic acid-producing strains can be divided into two categories, namely, the natural succinic acid producers (Table 2) and the metabolic engineering succinic acid producers (Table 3). The natural succinic acid producers mainly include *Actinobacillus succinogenes* (Thuy et al., 2017), *Anaerobiospirillum succiniciproducens* (Meynial-Salles et al., 2010), *Mannheimia succiniciproducens* (Lee et al., 2003), *Basfia succiniciproducens* (Choi et al., 2015) and *Escherichia coli* (Thakker et al., 2011). Among these strains, *A. succinogenes* and *A. succiniciproducens* were the first identified natural overproducers of succinic acid (Bretz and Kabasci, 2012). *A. succinogenes*, originally isolated from bovine rumen, is a Gram-negative, capnophilic, facultative anaerobic bacterium, and has been used as a succinic acid-producing chassis since 1981 by the Michigan Biotechnology Institute (MBI). *A. succinogenes* has a better tolerance of high concentrations of succinic acid than other strains (Wan et al., 2008) and can utilize extensive carbon sources, including glucose, xylose, glycerol, cellobiose, cheese whey (Wan et al., 2008), cane molasses (Liu et al., 2008), straw hydrolysate (Zheng et al., 2009), and crop stalk wastes. However, *A. succinogenes* also has some drawbacks as a succinic acid production platform, such as numerous auxotrophies and lack of useful genetic tools (Ahn et al., 2016). *A. succiniciproducens* is a Gram-negative and strictly anaerobic bacterium (Wang et al., 2011) that produces succinic acid and acetate as its major end products, while producing lactate and ethanol as minor products, depending on culture conditions (Lee et al., 1999). Compared with *A. succinogenes*, *A. succiniciproducens* shows only weak salt tolerance (Bretz and Kabasci, 2012), which partly hinders the wide application of this strain. *M. succiniciproducens*, also isolated from bovine rumen, is a facultatively anaerobic, mesophilic, non-motile, non-spore-forming, Gram-negative bacterium, and can utilize a wide variety of carbon sources, similar to *A. succinogenes* (Lee et al., 2002). This bacterium has an excellent CO_2_-fixing pathway and can produce succinic acid as a major end product through simple anaerobic fermentation. Nevertheless, this species also has some disadvantages, such as its pH sensitivity and auxotrophy for several amino acids and vitamins (Ahn et al., 2016). *B. succiniciproducens*, a new member of the family Pasteurellaceae, was isolated from the rumen of a German cow in 2008, and shows high similarity to *M. succinciproducens* (Ahn et al., 2016). In the 2,363 open reading frames (ORFs) of *B. succiniciproducens* DD1 and the 2,380 ORFs of *M. succiniciproducens* MBEL55 E, 2006 ORFs were found to be homologous (Kuhnert et al., 2010). This bacterium can export succinic acid as an end product and has been used as a succinic acid-producing chassis by the Succinity Company (a joint venture of BASF and Purac). Wild-type *E. coli* can also produce minor amounts of succinic acid under anaerobic conditions, whereas under aerobic conditions succinic acid is formed only as an intermediate of the TCA cycle unless the glyoxylate bypass is operating (Thakker et al., 2012). Table 2 shows a summary of bio-based succinic acid production by different wild-type strains with various fermentation substrates reported in selected papers. Among this list, *A. succinogenes*, *B. succiniciproducens*, and *M. succiniciproducens* are the most promising wild-type bacterial strains which have relatively high yield and productivity, but they still cannot satisfy industrial production requirements. Most wild-type strains show a tendency to degenerate (Lin et al., 2005b), pH sensitivity and auxotrophy, and they require a rich, complex medium for efficient growth. Thus, further optimization and improvement is needed before natural succinic acid producers can be used for industrial purposes.

**TABLE 2:** Production capacity of succinic acid by main natural microbes.

Strains	Substrate	Fermentation type	Titer (g/L)	Productivity (g/h/L)	Yield (g/g)	References
*A. succinogenes* FZ53	Glucose	Anaerobic batch	105.8	1.36	0.82	Pateraki et al. (2016)
*A. succinogenes* 130 Z	Cheese whey	Anaerobic batch	21.5	0.44	0.57	Wan et al. (2008)
*A. succinogenes* 130 Z	Glucose	Anaerobic batch	67.2	0.80	N/A	Guettler et al. (1996)
*A. succinogenes* 130 Z	Xylose	Anaerobic batch	38.4	0.94	0.70	Michael et al. (2015)
*A. succinogenes* CGMCC1593	Glucose	Anaerobic batch	60.2	1.30	0.75	Wang et al. (2011)
*A. succinogenes* CGMCC1593	Cane molasses	Anaerobic batch	50.6	0.84	0.80	Liu et al. (2008)
*A. succinogenes* NJ113	Glucose	Anaerobic batch	35.4	N/A	0.73	Chen et al. (2018)
*A. succinogenes* CGMCC1593	Straw hydrolysate	Anaerobic batch	45.5	0.19	0.81	Zheng et al. (2009)
*A. succinogenes* CGMCC2650	Cotton stalk	Anaerobic batch	15.8	0.62	1.23	Li et al. (2010)
*A.succiniciproducens* ATCC53488	Glucose	Anaerobicbatch	1.5	0.75	N/A	Meynial-Salles et al. (2010)
*M. succiniciproducens* MBEL55 E	Whey	Anaerobic batch	13.4	1.18	0.71	Lee et al. (2003)
*M. succiniciproducens* MBEL55 E	Glucose	Anaerobic batch	14	1.87	0.7	Lee et al. (2002)
*M. succiniciproducens* MBEL55 E	Wood hydrolysate	Anaerobic batch	11.73	1.17	0.56	Kim et al. (2004)
*B. succiniciproducens* DD1	Glucose	Anaerobic batch	20	0.68	0.49	Becker et al. (2013)
*E. coli*	Glucose	Anaerobic batch	1.18	0.13	0.12	Gokarn et al. (1998)
*C. glutamicum* R	Glucose	Micro-aerobic, fed-batch with membrane for cell recycling	23.0	3.63	0.19	Okino et al. (2005)

**TABLE 3:** Production capacity of succinic acid by metabolic engineering strains in selected papers.

Strains	Genotype	Substrate	Fermentation type	Titer (g/L)	Productivity (g/h/L)	Yield (g/g)	References
*E. coli* W1485	*ΔpflAB::Cm, ΔldhA::Kan, ΔptsG,* expression of *E. coli cra* gene with mutation at R57K, A58G, G59Q, R60Q, S75H, T76Y, D148I, R149I	Glucose	Two-stage fed-batch	79.8	1.00	0.78	Zhu et al. (2016)
*E. coli* W1485	*ΔackA-pta, ΔiclR, ΔpoxB, ΔmgsA, ΔsdhA::kan* ^*R*^ *,* expression of *C.glutamicum* ATCC13032 *pyc* gene	Glucose	Aerobic fed-batch	36.1	0.69	0.37	Yang et al. (2014)
*E. coli* DY329	*ΔackA, Δpta, ΔldhA, ΔpstG*, expression of *E. coli mdh* gene	Glucose	Two-stage fed-batch	32.3	0.40	-	Zhu et al. (2014)
*E. coli* SD121	Expression of *ppc*; deletion of *pflB*, *ldhA* and *pts*G	Glucose	Dual-phase fed- batch	116.2	1.55	1.13	Wang et al. (2011)
*E. coli* AFP111	Deletion of *pflB, ldhA* and *ptsG*	Glucose	Dual-phase fed- batch	101.2	1.89	1.07	Jiang et al. (2010)
*E. coli* MG1655	*ΔadhE, ΔldhA,* expression of *Lactococcus lactis pyc* gene	Glucose	Anaerobic fed-batch	15.6	0.65	0.85	Sánchez et al. (2005)
*E. coli* HL27659k (pKK313)	*ΔsdhAB, ΔackA-pta, ΔpoxB, ΔiclR, ΔptsG,* expression of *Sorghum vulgare pepc* gene	Glucose	Aerobic fed-batch	58.3	0.99	0.61	Lin et al. (2005d)
*E. coli* NZN111	Expression of *Ascaris suummaeA*gene	Glucose	Anaerobic shake flask	7.07	-	-	Stols et al. (1997)
*E. coli* JCL1208	lacks the *lac* operon but contains a chromosomally inserted*lacI* ^*q*^gene, expression of *E. coli ppc*under *tac* promoter	Glucose	Anaerobic batch	10.7	0.59	0.30	Millard et al. (1996)
*E. coli* K-12	*Δppc,* expression of *A. succinogenes pckA* gene	Glucose	Anaerobic batch	20.2	-	-	Millard et al. (1996)
*M. succiniciproducens* MBEL55 E	*ΔldhA::Km* ^*r*^ *, ΔpflB::Cm* ^*r*^ *, Δpta-ackA::Sp* ^*r*^	Glucose	Anaerobic fed-batch	52.4	1.80	1.16	Lee et al. (2006)
*M. succiniciproducens* PALK	*ΔldhA::Km* ^*r*^ *, Δpta-ackA::Sp* ^*r*^ *,* expression of *C. glutamicummdh*gene	Glucose	Two-stage fed-batch	134.3	21.3	0.81	Ahn et al. (2020)
*M. succiniciproducens* PALFK	Deletion of *ldhA, fruA* and *pta-ackA*	Sucrose and glycerol	Anaerobic fed-batch	78.4	6.02	1.07	Lee et al. (2016)
*M. succiniciproducens* LPK7	Expression of *fdh;* deletion of *ldhA, pflB* and *pta-ackA*	Sucrose and formic acid	Anaerobic fed-batch	76.1	4.08	-	Ahn et al. (2017)
*S. cerevisiaestrain* PMCFfg	*MATa ura3-52, Δhis3, Δfum1, Δgpd1, Δpdc1, Δpdc5, Δpdc6* (YIP-PYC2MDH3R, pRS313CF)	Glucose At PH 3.8	Aerobic batch	13.0	0.11	-	Yan et al. (2014)
*S.cerevisiae*CEN. PK 2-1C	*Δsdh2,* expression of *Rhizopus oryzaepyc* gene	Glucose	Anaerobic shake flask	0.8	0.01	-	Chen et al. (2019)
*Y. lipolytica* Y-3314	Expression of *pck, scs2;* deletion of *ach*	Glycerol	Aerobic fed-batch	110.7	0.80	0.53	Cui et al. (2017)
*Y. lipolytica* PGC01003	Deletion of *sdh5*	Glycerol	Aerobic fed-batch	198.2	-	-	Li et al. (2017)
*Y. lipolytica* Y-3314	Deletion of *sdh1*, *sdh2* and *suc2*	Glycerol	Aerobic fed-batch	45.4	0.28	0.36	Yuzbashev et al. (2010)
*C. glutamicum*ATCC 13032	*Δldh, Δpta-ackA, ΔactA, ΔpoxB, pyc* ^P458^ *, Δpck_*P_tuf_ *::Ms.pckG,* P_tuf_ *::ppc, ΔptsG*,expression of *C. glutamicum NCgl0275* gene	Glucose	Two-stage fed-batch	152.2	0.95	1.1	Chung et al. (2017)
*C. glutamicum*	Expression of *pyc;* deletion of *ldhA*	Glucose	Micro-aerobic fed- batch with membrane for cell recycling	146.0	3.17	0.92	Okino et al. (2008)
*C. glutamicum* BOL	Expression of *pyc, fdh* and *gapA;* deletion of *cat, pqo, ldhA* and *pta-ackA*	Glucose	Dual phase fed- batch	133.8	2.53	1.09	Litsanov et al. (2012)

Metabolic engineering strategies, however, is a promising way of generating succinic acid-producing strains, using not only natural succinic acid producers, but also unnatural producers, such as *Corynebacterium glutamicum, Yarrowia lipolytica, Saccharomyces cerevisiae, and Pichia kudriavzevii* (Table 3). Taking *C. glutamicum* as an example, the highest concentration of 152.2 g/L of succinic acid with a yield and productivity of 1.1 g/g glucose and 1.11 g/L/h, respectively, were achieved by the engineered strain S071 (*Δldh*, *Δpta-ackA*, *ΔactA*, *ΔpoxB*, *pyc* ^P458^, *Δpck*_P_tuf_:*Ms.pckG*, P_tuf_:*ppc*, *ΔptsG*)/pGEX4-NCgl0275 under anaerobic conditions. Compared with the initial strain, this engineered strain showed more than 30-fold increase in succinic acid production. (Chung et al., 2017). In the case of *Y. lipolytica*, the highest succinic acid concentration of 110.7 g/L was achieved, by the engineered strain *Y. lipolytica* PGC202 (*ΔSdh5*, *ΔAch1*, with *pck* from *S. cerevisiae* overexpression and *SCS2* from *Y. lipolytica* overexpression) in fed-batch cultivation using glycerol without pH control. Compared with the initial strain, this engineered strain showed 4.3-fold increase and has the highest fermentative succinic acid titer achieved in yeast at low pH condition (Cui et al., 2017). For *E. coli*, metabolic engineering strategies could be used to improve its succinic acid biosynthesis ability. Wu et al. constructed an engineered strain *E. coli* K12 (*Δpfl, ΔldhA, ΔptsG*)/pMD19T-*gadBC*. This strain could produce 32.01 g/L succinic acid at pH 5.6, which was 2.6-fold higher than the initial strain (Wu et al., 2017). These metabolic engineering producers have shown high production efficiency, demonstrating the great potential of the production of industrially competitive bio-based succinic acid.

In this review, several different typical succinic acid biosynthetic pathways reported in recent years are summarized, and the main metabolic engineering strategies to improve the production efficiency are presented in detail. Additionally, the main challenges as well as the future perspectives for succinic acid biosynthesis are discussed.

## 2 Biosynthetic Pathway of Succinic Acid

**FIGURE 1:**
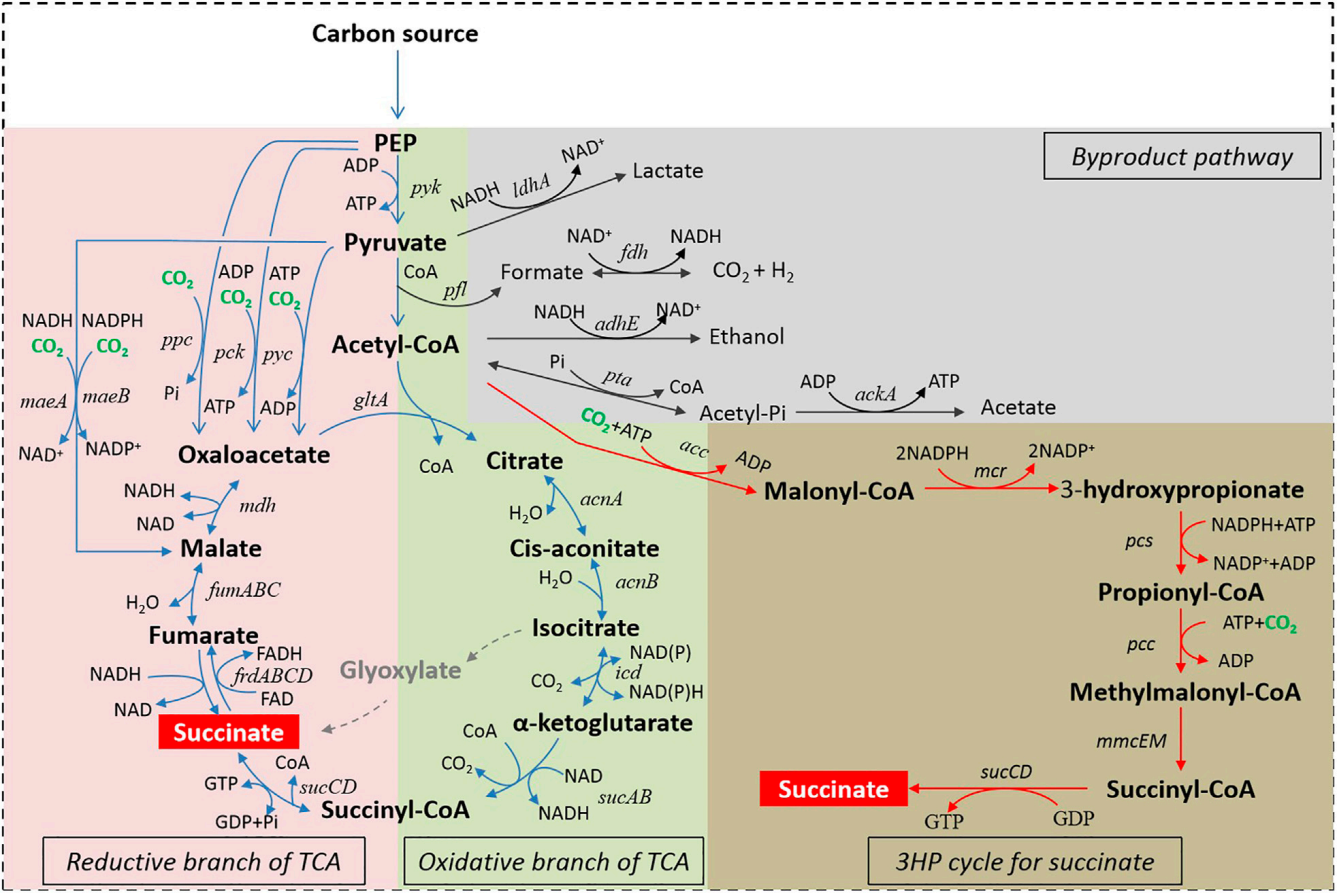
Succinic acid production biosynthetic pathways. Abbreviations: PEP, Phosphoenolpyruvic acid; *ldhA*, lactic dehydrogenase; *pfl*, pyruvate formate lyase; *fdh*, formate dehydrogenase; *adhE*, alcohol dehydrogenase; *pta*, phosphotransacetylase; *ackA*, acetate kinase; *gltA*, citrate synthetase; *acnAB*, aconitase; *icd*, isocitrate dehydrogenase; *sucABCD*, succinyl-CoA synthetase; *frdABCD*, succinate dehydrogenase; *fumABC*, fumarate hydratase; *mdh*, malate dehydrogenase; *ppc*, PEP carboxylase; *pck,* PEP carboxykinase; *pyc*, pyruvate carboxylase; *pyk*, pyruvate kinase; *maeAB*, malic enzyme; *acc*, acetyl-CoA carboxylase; *mcr*, malonyl-CoA reductase; *pcc*, propionyl-CoA carboxylase; *pcs*, propionyl-CoA synthase; *mmcEM*, methylmalonyl-CoA epimerase and mutase.

To date, seven natural or artificial succinic acid biosynthesis pathways have been reported in the literature (Figure 1), including the reductive branch of the TCA cycle coupled to PEP carboxylase (PPC) pathway, the reductive branch of the TCA cycle coupled to PEP carboxykinase (PCK) pathway, the reductive branch of the TCA cycle coupled to pyruvate carboxylase (PYC) pathway, the reductive branch of the TCA cycle coupled to malic enzyme (MAE) pathway, the glyoxylate shunt pathway, the oxidative branch pathway of the TCA cycle, and the 3-hydroxypropionate cycle for the production of succinic acid. Each biosynthesis pathway has its own characteristics that determine the scope of their unique application (Table 1). The reductive branch pathways of the TCA cycle are the main succinic acid-producing approaches under anaerobic conditions (Zhu and Tang, 2017), while the other pathways can be effective under aerobic conditions. In the next subsections, these different pathways and their specific features are discussed.

### 2.1 Anaerobic Succinic Acid-Producing Pathways

The reductive branch of the TCA cycle coupled to PEP carboxylase (PPC) for succinic acid formation (rTCA (PPC) pathway), which exists in almost all microbes, is one of the most commonly used processes in bio-based succinic acid production. In this pathway, PEP carboxylase (PPC) converts PEP and CO_2_ into oxaloacetate, which is then converted into malate catalyzed by malate dehydrogenase (MDH). Then, malate is transformed into fumarate by fumarate hydratase (FumABC). Finally, fumarate and NADH are turned into succinic acid catalyzed by fumarate reductase (FrdABCD). Through this pathway, succinic acid is synthesized from PEP and CO_2_ at a stoichiometric ratio of 1:1. Among the components in this process, PPC (EC 4.1.1.31) plays a critical role in CO_2_ fixation. Taking *E. coli* as an example, PPC is produced during growth on glycolytic substrates (Keseler et al., 2011). PPC catalyzes the carboxylation of PEP with bicarbonate to form oxaloacetate and inorganic phosphate using Mg^2+^ as a cofactor (Millard et al., 1996; Kai et al., 1999). The main function of PPC is to replenish the oxaloacetate consumed by biosynthetic reactions, and under anaerobic fermentation conditions, it can direct a portion of PEP to succinic acid (Millard et al., 1996). Studies have shown that under a CO_2_ atmosphere, *E. coli Δppc* strains grow slower and more poorly than wild-type strains in glucose-based medium (40% reduction in biomass and more than 70% reduction in succinic acid production) (Millard et al., 1996; Ahn et al., 2020). PPC also exhibits high catalytic velocity (the enzymatic activity is 250 μmol/min/mg) and high substrate affinity (the *K*_m_ value for HCO_3_^-^ is 0.1 mM and the *K*_m_ value for PEP is 0.19 mM), which is conducive to succinic acid biosynthesis (Millard et al., 1996; Kai et al., 1999; Tan et al., 2013). Millard et al. evaluated the effect of PPC on the amount of succinic acid produced in *E. coli* under control of the *tac* promoter. The engineered strain that overexpressed PPC showed a 3.5-fold increase in the concentration of succinic acid, from 3.0 g/L in a control culture to 10.7 g/L, and the yield showed a 3.75-fold increase, from 0.12 mol/mol glucose to 0.45 mol/mol glucose (Table 3) (Millard et al., 1996). There seemed to be a positive correlation between PPC activity and succinic acid production, but excessive PPC activity was detrimental to cell growth and succinic acid formation (Tan et al., 2013). Tan et al. found higher PPC activity along with higher succinic acid production, when the PPC activity was equal to or less than 0.47 U/mg protein (Tan et al., 2013). The reason may have been that the overexpression of PPC caused a shortage of PEP. PEP is also essential for the PEP-PTS (phosphoenolpyruvate-phosphotransferase) system, which has both a transport function and an extensive regulatory function. And the shortage of PEP will lead to a compromise in glucose uptake and cell growth (Gokarn et al., 2000). Therefore, the regulation of PPC expression was significant when using the PPC dependent biosynthesis pathway for succinic acid production.

The reductive branch of the TCA cycle coupled to PEP carboxykinase (PCK) for succinic acid formation (rTCA (PCK) pathway), is another representative succinic acid biosynthesis route that mainly exists in bacteria and fungi, especially in some natural succinic acid producers (Werf et al., 1997; Lee et al., 2006). This process is almost the same as the PPC-dependent pathway, except for the key CO_2_-fixation enzyme. In this pathway, the key CO_2_-fixation enzyme is PCK (EC 4.1.1.31) which can catalyze the carboxylation of PEP with bicarbonate and ADP to form oxaloacetate and ATP. Compared with PPC, the PCK catalytic process is accompanied by the production of energy, and as such it is more suitable for cell growth and succinic acid production (Zhu and Tang, 2017). However, the overexpression of PCK in *E. coli* at low CO_2_ concentrations in fact has no effect on succinic acid biosynthesis. PCK can be valuable for succinic acid production but only in the presence of high HCO_3_^-^ concentrations (Millard et al., 1996; Kwon et al., 2006). There are two possible explanations for this result: 1) PCK has low HCO_3_^-^ affinity (the *K*_m_ value for HCO_3_^-^ is 13 mM, 130 times larger than that of PPC, the *K*m value for PEP is 0.07 mM and the *K*m value for ADP is 0.05 mM); 2) PCK has low catalytic velocity (the enzymatic activity is 28 μmol/min/mg, which is just 11.2% of the PPC activity) (Krebs and Bridger, 1980; Zhang et al., 2009a; Tan et al., 2013). Previous studies have demonstrated that when 20 g/L NaHCO_3_ was added to a culture, the succinic acid titer in recombinant *E. coli* overexpressing PCK was 2.2-fold higher than a wild-type strain, from 6.4 g/L in a control culture to 14.2 g/L (Kwon et al., 2006). Kim et al. found that in wild-type *E. coli* K12 cells, PCK overexpression had no effect on succinic acid fermentation, but the overexpression of PCK in an engineered *E. coli* K12 *Δppc* strain was 6.5-fold higher than *E. coli* K12 *Δppc*, and rose from 3.1 g/L to 20.2 g/L (Table 3) (Millard et al., 1996). In addition, the PCK activity was closely related to succinic acid biosynthesis. Tan et al. reported that the succinic acid titer and yield were positively correlated with the PCK activity. A 16-fold increase in PCK activity resulted in a 57-fold increase in succinic acid titer (from 4 to 226 mM) in *E. coli* Suc-T108 (Tan et al., 2013). In some natural succinic acid producers, such as *A. succiniciproducens* (Millard et al., 1996)*, M. succiniciproducens* (Hong et al., 2004; Lee et al., 2006)*,* and *A. succinogenes* (Millard et al., 1996; Werf et al., 1997), PCK is mainly employed in CO_2_ fixation and cell growth instead of PPC. Taking *M. succiniciproducens* as an example, genes encoding PPC, PCK, or MAE were separately disrupted, and fermentation results showed that strains with PPC or MAE deletion did not show much effect on succinic acid productivity or cell growth, while the strain with PCK inactivation showed severe growth retardation and a remarkable decrease in succinic acid productivity (Lee et al., 2006). This result was significant because although PCK has a high *K*_m_ value for HCO_3_^-^, it still plays a major role in succinic acid biosynthesis in *M. succiniciproducens*. Compared with *M. succiniciproducens*, the indispensable feature of PCK was not observed in wild-type *E. coli*. It might have been due to the differences in the internal concentration of HCO_3_^-^ in these two organisms (Millard et al., 1996). These results also suggests that the optimization of a CO_2_ delivery system and the amelioration of the intracellular inorganic carbon concentration may be a good choice for improving the PCK depended succinic acid biosynthesis route (Xiao et al., 2017). Except for PCK, MDH was reported to be another rate-limiting enzyme for succinic acid production (Hong et al., 2004; Ahn et al., 2020). In *M. succiniciproducens*, MDH reduces oxaloacetate to malate using NADH as a cofactor, but it shows low specific activity and strong uncompetitive inhibition towards oxaloacetate (Ahn et al., 2020). *In silico* simulation suggested that MDH is instrumental in succinic acid production (Ahn et al., 2020). Jung et al. reported biochemical and structural analyses of various MDH variants and discovered a key amino acid residue (Gly-11) required for high kinetic efficiency in MDH. After screening, a valuable MDH mutant (MDH-G11Q) was obtained, which had high enzyme activity (2.9-fold increase in activity) and less substrate inhibition (6.4-fold increase in *k*_*i*_) compared with a wild type MDH. Furthermore, the application of this MDH-G11Q mutant in engineering bacteria led to a 1.4-fold increase in succinic acid production, indicating the significance of enzyme optimization in strain development (Choi et al., 2016; Ahn et al., 2020).

The reductive branch of the TCA cycle coupled to pyruvate carboxylase (PYC) for succinic acid formation (rTCA (PYC) pathway), is widespread in fungi, plants, as well as some bacteria (Modak and Kelly, 1995), but it is absent in Enterobacteriaceae (Gokarn et al., 2000). In this pathway, PEP is first converted into pyruvate by pyruvate kinase (PYK). Then PYC (EC 6.4.1.1) catalyzes the ATP-dependent carboxylation of pyruvate to produce oxaloacetate. Oxaloacetate is further converted into succinic acid through MDH, FumABC and FrdABCD. As the key CO_2_-fixation step, the reaction catalyzed by PYC also plays an anaplerotic role in the provision of oxaloacetate for maintaining the dynamic balance of the TCA cycle. In *R. etli*, the anaplerotic role of PYC has been shown to be essential for its normal growth (Jitrapakdee et al., 2008), and thus PYC can be considered to be one of the most significant regulatory enzymes in this pathway (Modak and Kelly, 1995). In most PYC catalytic reactions, acetyl-CoA acts as a positive allosteric activator (Jitrapakdee et al., 2008; Tong, 2013), except for PYC from *L. lactis*, which is regulated by the bacterial second messenger c-di-AMP (Choi et al., 2017). The PYC depended route is a promising tool for succinic acid biosynthesis. Yan et al. reconstructed this pathway in *S. cerevisiae*, and found that the engineered strain showed a 19.3-fold increase in succinic acid production in shaken flasks, rising from 0.32 g/L to 6.17 g/L (Yan et al., 2014). Chen et al. reported that the overexpression of PYC from *R. oryzae* in a *S. cerevisiaeΔsdh2* strain resulted in a 1.2-fold increase in the concentration of succinic acid, from 0.709 g/L to 0.841 g/L (Table 3) (Chen et al., 2019). In addition, the functions of PYC, PPC and MAE on the carboxylation processes for high succinic acid productivity in *E. coli* were analyzed *in silico*. According to analysis results, carboxylation reactions catalyzed by PYC were the most suitable ones for obtain high productivity in *E. coli*. Furthermore, based on these analysis results, PYC from *C. glutamicum* ATCC13032 was overexpressed in *E. coli* ZJG13 and the final succinic acid production was 36.1 g/L, with a specific productivity and yield of 2.75 mmol g CDW^−1^ h^−1^ and 0.72 mol mol^−1^ glucose, respectively (Table 3) (Yang et al., 2014). Sánchez et al. overexpressed the *L. lactis pyc* gene in *E. coli* MG1655 (*ΔadhE, ΔldhA*) and found that a 25-fold increasein succinic acid production, from 0.6 g/L to 15.6 g/L, as well as a 6-fold increase in the yield, from 0.13 g/g glucose to 0.78 g/g glucose (Table 3) (Sánchez et al., 2005).

The reductive branch of the TCA cycle coupled to malic enzyme (MAE) for succinic acid formation (rTCA (MAE) pathway), is shorter than the PYC dependent pathway. In the MAE dependent pathway, pyruvate and CO_2_ are directly converted into malate by malic enzyme, and malate is further converted into succinic acid through FumABC and FrdABCD. There are two forms of malic enzymes: MaeA (EC 1.1.1.38) and MaeB (EC 1.1.1.40), which can use NADH and NADPH as electron donors, respectively. Stols et al. overexpressed MAE from *A. suum* in *E. coli* NZN111 to produce succinic acid, resulting in a 2.9-fold increase, from 2.45 g/L to 7.07 g/L (Table 3) (Stols et al., 1997). Kwon et al. cloned MaeA and MaeB, derived from *E. coli* W3110, and overexpressed them in *E. coli* W3110 under anaerobic conditions. They found that the enzyme activities of overexpressed MaeA and MaeB were 2.34 and 1.69 μmol min^−1^mg_protein_^−1^, whereas their activities were only 0.82 and 0.01 μmol min^−1^mg_protein_^−1^ in the wild-type W3110 strain, respectively (Kwon et al., 2007). Furthermore, the succinic acid yields were measured in different strains. MaeB overexpression resulted in a 2.4-fold increase in succinic acid production, to 15.43 mmol/L, compared with 6.41 mmol/L in wild-type *E. coli* W3110. However, MaeA overexpression resulted in just 6.69 mmol/L succinic acid, which was similar to a wild-type strain under the same anaerobic fermentation conditions (Kwon et al., 2007). In this study, there was a negative correlation between succinic acid production and the activities of MaeA and MaeB. The main reason for this result might have been related to the differences in the intracellular co-substrate availability of MaeA and MaeB. The intracellular concentration of NADPH was 146 μmol/L, which was 9.12-fold higher than the 16 μmol/L of NADH intracellular concentration (Bochner and Ames, 1982; Kwon et al., 2007). Correspondingly, NADPH-dependent MaeB showed an advantage over the NADH-dependent MaeA, which would encourage CO_2_ fixation by MaeB over by MaeA in wild-type *E. coli* (Kwon et al., 2007). In addition, the reaction catalyzed by MAE is reversible and this enzyme prefers to form pyruvate, which is unfavorable for succinic acid formation (Hong and Lee, 2002). The *K*_m_ values of MAE for malate and pyruvate in *E. coli* are 0.4 and 16 mM, respectively. Similarly, the *K*_m_ values of MAE in *C. glutamicum*are 3.8 mM for malate and 13.8 mM for pyruvate, respectively (Stols and Donnelly, 1997; Gourdon et al., 2000). This indicates that sufficiently high intracellular pyruvate concentrations may have a significant influence to overcome the unfavorable equilibrium of MAE catalytic processes. However, the wild-type strain is unable to accumulate a sufficiently high pyruvate concentration to allow MAE to be effective for producing excessive malate (Stols et al., 1997). Therefore, the enhancement of the PYK catalytic process, which can convert PEP and ADP into pyruvate and ATP, maybe a good choice for intracellular pyruvate enrichment. In some natural succinic acid producers, this reaction is also closely related to normal metabolic activity. Taking *M. succiniciproducens* as an example, Lee found that PYK operates by providing the pyruvate and ATP required for cell growth, as its pyruvate concentration increases with cell growth and remains constant after cell growth has stopped (Lee et al., 2006).

### 2.2 Aerobic Succinic Acid-Producing Pathways

Although high productivities and yields of succinic acid can be achieved through anaerobic biosynthesis pathways, some drawbacks still exist with using these pathways, such as their slow carbon throughput, limitation of NADH availability (2 mol of NADH are required for 1 mol succinic acid formation) and poor cell growth (Yang et al., 2014). One solution is to produce succinic acid under aerobic conditions, which can generate higher biomass and extensive energy with O_2_ as the electron acceptor (Lin et al., 2005a; 2005b; Yang et al., 2014). The oxidative branch pathway of the TCA cycle (oTCA pathway) is one of the candidate aerobic biosynthesis routes for succinic acid production. In this pathway, acetyl-CoA and oxaloacetate are converted into succinic acid through citrate synthetase (GltA), aconitase (AcnAB), isocitrate dehydrogenase (ICD), and Succinyl-CoA synthetase (SucABCD), accompanied by the synthesis of NADH at a stoichiometric ratio of 2:1. Although succinic acid can be generated by oTCA under aerobic conditions, most of it will be further converted in the TCA cycle or to biomass and cannot be excreted from the cells. Thus, the metabolic engineering studies are necessary to improve its yield. Taking *E. coli* as an example, succinic acid is a minor fermentation product under anaerobic conditions. Under aerobic conditions, however, succinic acid is only an intermediate of the TCA cycle and cannot be detected in the extracellular medium (Clark, 1989; Lin et al., 2005b). Correspondingly, acetate is the main byproduct under the same aerobic conditions (Lin et al., 2005b). In order to produce succinic acid as a major product aerobically, Lin et al. created a mutant *E. coli* HL27659k (pKK313) strain that overexpressed of *S. vulgare pepc* and had five enzymes inactivated: *ΔsdhAB, Δ(ackA-pta), ΔpoxB, ΔiclR, andΔptsG.* This mutant strain could use both the oxidative branch pathway of the TCA cycle and the glyoxylate shunt pathway to produce succinic acid under aerobic conditions, and its maximum theoretical succinic acid yield could reach 1.0 mol/mol glucose consumed (Lin et al., 2005c). Fed-batch fermentation results showed that this strain could produce 58.3 g/L of succinic acid under complete aerobic conditions with a yield of 0.94 mol/mol glucose consumed (Table 3) (Lin et al., 2005b; 2005d). Arikawa et al. constructed a *Saccharomyces cerevisiae* mutant with fumarate reductase (FRDS) and succinate dehydrogenase (SDH1) inactivated. However, under aerobic conditions, this mutant strain could synthesize succinic acid through the oxidative branch pathway of the TCA cycle and the succinic acid yield showed a 2.7-fold increase compared with the parental strain (Arikawa et al., 1998). *S. cerevisiae* exhibits a high tolerance to low pH values, which makes it superior for succinic acid production (Qiang et al., 2010). These results were significant and Lin et al. inferred the potential of the oTCA pathway for aerobic succinic acid biosynthesis (Lin et al., 2005b).

The glyoxylate shunt pathway (GAC pathway) is another aerobic succinic acid biosynthesis route. In this process, 2 mol of acetyl-CoA and 1 mol of oxalacetate are converted into 1 mol of malate and 1 mol of succinic acid by citrate synthetase (GltA), aconitase (AcnAB) and isocitrate lyase (AceAB). Compared with the oxidative branch pathway of the TCA cycle, this process bypasses the two oxidative steps where CO_2_ is released, and thus it is considered to be an atom-economic aerobic pathway (Lin et al., 2004). Additionally, malate is produced as the main byproduct in the glyoxylate shunt pathway. Malate can be catalyzed by malate dehydrogenase (MDH) to generate NADH and regeneration of oxaloacetate. Under anaerobic conditions, 1 mol of malate can also be further transformed into 1 mol of succinic acid by FumABC and FrdABCD by consuming 1 mol of NADH. Compared with the traditional rTCA pathway (formation of 1 mol of succinic acid requires 2 mol of NADH), the glyoxylate shunt pathway does not contain NADH consumption process. Therefore, its application will be beneficial for solving the limitation of NADH availability during succinic acid biosynthesis (Sánchez et al., 2005). Vemuri et al. found that in the absence of an additional electron acceptor under dual-phase conditions (an aerobic growth phase followed by an anaerobic production phase), the maximum theoretical succinic acid yield was up to 1.714 mol/mol glucose, when 28.6% of the carbon flows to the glyoxylate shunt pathway and 71.4% of the carbon flows to the reductive branch pathway of the TCA cycle. It is worth noting that the maximal yield of succinic acid cannot be achieved without an active glyoxylate shunt pathway (Vemuri et al., 2002). It should be noticed that C2 substrates like acetate or fatty acids are very prominent activators for the glyoxylate shunt pathway under aerobic conditions (Gui et al., 1996). The *aceBAK* operon, which encodes enzymes of the glyoxylate cycle, is controlled by IclR. Under aerobic conditions, acetate can induce *aceBAK* and reduce the repressor activity of IclR, resulting in activation of the glyoxylate cycle (Gui et al., 1996; Sánchez et al., 2005). Meanwhile, studies have also revealed that the disruption of *iclR* could dramatically induce the expression of the *aceBAK* operon even when growing on glucose (Gui et al., 1996; Lin et al., 2004; Hendrik et al., 2011). Thus, the inactivation of *iclR* is significant for activating the glyoxylate shunt pathway for succinic acid production (Sánchez et al., 2005). Lin et al. constructed an engineered *E. coli* HL27615k strain with mutations in the TCA cycle (*ΔiclR, Δicd,* and *ΔsdhAB*) and acetate pathways (*ΔpoxB,* and *ΔackA-pta*) for maximal aerobic succinic acid production through the glyoxylate shunt pathway. The results of aerobic batch fermentation showed that the succinic acid production reached 43 mM with a yield of 0.7, which was close to the maximum theoretical yield of 1 mol/mol glucose (Lin et al., 2004). Except for the absence of the *iclR* mutation, Zhu et al. demonstrated that the glyoxylate shunt pathway could also be activated by deleting other relevant genes in *E. coli* under anaerobic conditions. The key genes included *ackA* (a gene encoding an acetate kinase) and *pta* (a gene encoding a phosphotransacetylase). Metabolic flux analysis reflected that the carbon flow shunted to the glyoxylate pathway from oxalacetate in wild-type *E. coli* DY329 was 0 and 31% in the mutant YJ003 (*ΔackA, Δpta, ΔldhA,* and *ΔpstG*). In addition, the succinic acid production also showed a 6-fold increase, from 25.13 mM in DY329 to 150.78 mM in YJ003, implying the importance of the activation of the glyoxylate shunt pathway (Zhu et al., 2014).

The 3-hydroxypropionate cycle is one of the natural aerobic CO_2_-fixation pathways, and mainly exists in photosynthetic green nonsulfur bacteria (Herter et al., 2001). This cycle is complex, containing 16 enzymatic reaction steps that are catalyzed by 13 enzymes (Gong et al., 2016). Liu et al. introduced part of this cycle into recombinant *E. coli* for succinic acid production (3HP pathway) (Liu et al., 2020). In this process, acetyl-CoA is carboxylated by acetyl-CoA carboxylase (ACC) to generate malony-CoA. Malony-CoA is converted into propionyl-CoA by malonyl-CoA reductase (MCR) and propionyl-CoA synthase (PCS). Then propionyl-CoA is carboxylated by propionyl-CoA carboxylase (PCC) to generate (*S*)-methylmalonyl-CoA. Next, methylmalonyl-CoA epimerase (MmcE) and methylmalonyl-CoA mutase (MmcM) convert (*S*)-methylmalonyl-CoA into its isomer succinyl-CoA. Finally, succinyl-CoA is deesterificated by succinyl-CoA synthetase (SucCD) to generate succinic acid (Menendez et al., 1999; Liu et al., 2020). The conversion of 1 mol of acetyl-CoA into 1 mol of succinic acid via the 3-hydroxypropionate cycle can fix 2 mol of CO_2_, which was confirmed by isotope labeling experiments with NaH^13^CO_3_ (Liu et al., 2020). Compared with succinic acid production based on carboxylation of PEP or pyruvate, this route showed a higher CO_2_ fixation efficiency. Among these pathway, ACC (EC 6.4.1.2) and PCC (EC 6.4.1.3) are the key CO_2_-fixing and rate-limiting enzymes. Both ACC and PCC belong to a subgroup of biotin-dependent short-chain acyl-CoA carboxylases that contain biotin carboxyl carrier protein domain (BCCP), biotin carboxylase domain (BC), and carboxyltransferase domain (CT), and utilize a covalently bound biotin as a cofactor (Wongkittichote et al., 2017; Liu and Jiang, 2021). The CT domain determines the specificity of the substrate, and these proteins can even work with other BCCP and BC domains from different enzymes (Lombard and Moreira, 2012; Tong, 2013). To obtain PCC with high enzymatic activity, Liu et al. tested different CT subunits of the PCC homologs from diverse bacterial species and developed a direct evolution strategy to further optimize this protein. A highly active PCC mutant (PccB_BS_-N220I/I391T) was obtained, which showed a 94-fold increase in overall catalytic efficiency indicated by *k*_cat_/*K*_m_ compared with a wild-type PCC. In addition, the application of this PCC mutant resulted in a 1.5-fold increase in succinic acid production compared to the engineered strain with wild-type PCC, which indicated the significance of this rate-limiting enzyme engineering strategy for microbial production (Liu et al., 2020). ACC acts as another CO_2_-fixing enzyme and ACC is essential for most living organism’s growth (Baba et al., 2006). The intracellular concentration of its carboxylation product (malonyl-CoA) is tightly regulated to be very low, leading to limited production of ACC-derived compounds (Zha et al., 2009; Liu et al., 2010). It has been reported that ACC is negatively regulated by AMP-activated serine/threonine protein kinase (Snf1) in *S. cerevisiae* when glucose is depleted. Moreover, the phosphorylation triggered by Snf1 at one or more serine residues results in the deactivation of ACC (Woods et al., 1994; Scott et al., 2002). Jin et al. identified a critical amino acid (Ser-1157) responsible for deactivation via phosphorylation and mutated it to an alanine. The *in vitro* activity results showed that this ACC mutant (ACC-S1157A) resulted in 9-fold higher specific activity relative to wild-type ACC (Choi and Silva, 2014). Shi et al. demonstrated that introduction of this S659A mutation in ACC-S1157A could also lead to an enhanced enzyme activity with 1.72-fold increase (Shi et al., 2014). Further utilization of these modified acetyl-CoA carboxylase will be beneficial for product biosynthesis built from malonyl-CoA, such as succinic acid (Choi and Silva, 2014). Except for ACC and PCC, malonyl-CoA reductase (MCR), a characteristic enzyme of the 3-hydroxypropionate cycle, is regarded as another rate-limiting enzyme (Fixation et al., 2002). MCR catalyzes a two-step NADPH-dependent reduction of malonyl-CoA to 3-hydroxypropionate, and malonate semialdehyde has been suggested to be the likely free intermediate (Fixation et al., 2002; Liu et al., 2013). Liu et al. demonstrated that MCR, a natural fusion protein of two short-chain dehydrogenase/reductases, has higher enzyme activity when dissected into two functionally individual fragments, including an MCR-C fragment (amino acids 550–1,219) and MCR-N fragment (amino acids 1–549) (Liu et al., 2013). Further studies indicated that the initial reduction of malonyl-CoA to malonate semialdehyde catalyzed by MCR-C was the rate-limiting step of MCR (Liu et al., 2013; Liu et al., 2016). Liu et al. developed a direct evolution strategy to further optimize MCR-C. A triple MCR-C mutant (N940V/K1106W/S1114R) was obtained, which showed a 5.54-fold increase in enzyme activity. Combined with fine tuning of the MCR-N expression levels and culture condition optimization, the 3-hydroxypropionate yield showed a 270-fold increase, which could relieve the pressure of being the rate-limiting step in succinic acid biosynthesis (Liu et al., 2016). Although some progress has been achieved in this succinic acid pathway, some problems are still worthy of attention. The main challenge is that significant amounts of ATP and reducing power (3ATP and 3NADPH) are consumed from one molecule acetyl-CoA to one molecule succinic acid. Using fatty acids as carbon sources or feeding electricity to bacteria to produce intracellular energy may be optional promising approaches (Chen et al., 2018; Liu et al., 2019).

## 3 Metabolic Engineering for Enhancing Succinic Acid Biosynthesis

Up to now, the bio-based succinic acid synthetic process has become increasingly mature. There are several strategies to enhance succinic acid biosynthesis, which mainly include redirecting carbon flux, balancing the redox ratio (NADH/NAD^+^) and optimizing CO_2_ supplementation. And the different strategies will be discussed in more details in the later chapters.

### 3.1 Redirecting Carbon Flux

Whether under anaerobic or aerobic conditions, succinic acid is not the main product of many microbes. In general, microbes preferentially accumulate substances such as acetate, lactate, and ethanol etc. These by-product pathways compete with the succinic acid pathway for ATP or reducing power. Therefore, it is necessary to redirect the carbon flux from these competitive pathways into succinic acid production (Li et al., 2020). The redirection methods often used mainly include blocking these competitive pathways and regulating succinic acid biosynthesis pathways.

#### 3.1.1 Blocking Competitive Pathways

As the main competing by-products, acetate, lactate, and ethanol are the targets of metabolic regulation, and deletion of the genes related to their production is usually performed. Specifically, single deletion of *ldhA* (encoding lactate dehydrogenase), which can convert pyruvate and NADH into lactate and NAD^+^ in *E. coli*, led to a 1.6-fold increase in succinic acid yield under anaerobic conditions, from 0.13 to 0.21 mol/mol glucose. Meanwhile, the lactate concentration of this *ΔldhA* mutant was 92% lower than a wild-type strain, dropping from 214 to 17 mM (Zhang et al., 2009b). Single deletion of *adhE* (encoding alcohol dehydrogenase), which converts acetyl-CoA and NADH into ethanol and NAD^+^, resulted in a 1.125-fold increase in succinic acid yield. Correspondingly, the ethanol concentration of this *ΔadhE* mutant was 0, compared with 133 mM in a wild-type strain (Zhang et al., 2009b). Zhang et al. constructed an *E. coli ΔldhAΔadhE* double mutant strain, which showed a 1.77-fold increase in succinic acid yield, and lactate and ethanol were not detected in its fermentation broth (Zhang et al., 2009b). There are two major acetate-producing pathways in *E. coli*, respectively through the enzyme pyruvate oxidase (encoded by *poxB*) and acetate kinase/phosphotransacetylase (encoded by *ackA-pta*). Pyruvate oxidase catalyzes the decarboxylation of pyruvate to acetate and CO_2_, and is more active in the stationary stage of cell growth (Mey et al., 2007; Dittrich, 2010). Ahmed et al. demonstrated that PoxB was useful for overall metabolism functioning and single deletion of *poxB* led to a decrease of 24% in biomass. Thus, they suggested that employing *poxB* served as a method to decrease acetate production (Abdel-Hamid et al., 2001). The *ackA-pta* pathway, which is more active in the exponential phases of cell growth, involves two consecutive reactions. PTA converts acetyl-CoA and Pi into acetyl phosphate and CoA. AckA then converts acetyl phosphate and ADP into acetate and ATP (Zhu et al., 2014). It was demonstrated that *pta* mutants grow more slowly than wild-type strains. The specific rate of growth of *E. coli* MG1655 Δ*pta* was 9.3% lower than the parent strain under aerobic conditions and 37.2% lower under anaerobic conditions (Schütze et al., 2020). This may have been due to pyruvate accumulation, acetyl-P accumulation or a redox imbalance in *pta* mutants (Wolfe, 2005). Wolfe reported that *pta* mutants or *ackA-pta* mutants did not accumulate extracellular acetate, and *ackA* mutants could accumulate small amounts of acetate (Wolfe, 2005). Compared to a particular mutation, the combination of mutations was proved more useful for enhancing succinic acid production in many cases. Zhang et al. constructed a *ΔldhAΔadhEΔackA* triple mutant, which showed a 3.2-fold increase in succinic acid concentration and a 5.4-fold increase in yield (Zhang et al., 2009b). Lu et al. constructed a *E. coli* JH208 (*ΔldhA*, *ΔpflB*, *ΔadhE, ΔpoxB, ΔackA, ΔcscR*) mutant strain, and found that this mutant could produce 48.46 g/L succinic acid in 46 h with the productivity of 1.01 g/L/h and the yield of 0.83 g/g sucrose. Compared with the initial strain, the production efficiency of succinic acid has been improved 72% (Lu et al., 2015). Olajuyin et al. constructed an *E. coli* K-12 (*ΔldhA*, *ΔpflB*, *ΔpoxB, Δpta-ackA*) mutant for succinic acid production, the concentration and molar yield of succinic acid were respectively 22.40 ± 0.12 g/L and 1.13 ± 0.02 mol/mol total sugar after 72 h dual phase fermentation, and the final concentration of succinic acid was 6.2-fold higher than the wild-type strain (Olajuyin et al., 2016). These results indicated the necessity of blocking competitive pathways for improving succinic acid yield.

#### 3.1.2 Regulating Succinic Acid Pathways

Direct overexpression of important enzymes (mentioned in Section 2) is the most frequently used method for enhancing the production of the succinic acid pathways. However, biosynthetic processes are usually regulated by multiple genes, and positive expected results are difficult to obtain by simple genetic engineering. In recent years, investigation of the global regulation of gene expression has been shown to be effective for solving the limitations of classical metabolic engineering approaches (Chong et al., 2014; Zhu and Tang, 2017). Some global regulators are good targets for improving the succinic acid biosynthesis process, such as ArcA, Crp, Cya and Cra. Among them, the Arc (anoxic respiration control) system composed of ArcA and ArcB regulates gene expression in response to redox conditions (Liu and Wulf, 2004; Sun et al., 2020). As reported, ArcA, when phosphorylated, represses the expressions of the genes involved in the TCA cycle and the glyoxylate shunt genes (Shimizu, 2013). Knocking out of ArcA did not affect the growth rate and the glucose uptake rate, but increased the TCA cycle activity by over 60% under aerobic conditions. It is significant for the oTCA pathway and the glyoxylate shunt pathway (Perrenoud and Sauer, 2005). Crp (cyclic AMP receptor protein) is the catabolite repressor that is activated by adenylate cyclase (Cya)-synthesized cAMP (Zhang et al., 2014). Crp/cAMP is a positive transcriptional regulator for *mdh, ptsG, pckA, sdhABCD, acnAB, gltA* (Perrenoud and Sauer, 2005; Wilfredo et al., 2021). Research showed that knocking out of Crp or Cya would decrease the growth rate, the glucose consumption rate and reduce the metabolic flux from PEP to oxaloacetate (Perrenoud and Sauer, 2005). Catabolite repressor/activator (Cra), also known as FruR, is an important global transcription factor, and it plays a key role in balancing the levels of the genes involved in carbon metabolism in *E. coli* (Akira, 2000; Ishihama, 2010; Shimada et al., 2011). Cra can activate genes related to the tricarboxylic acid cycle, the glyoxylate shunt pathway (Cozzone and El-Mansi, 2005; Dayanidhi et al., 2008; Shimada et al., 2011), and negatively affects genes related with the ED and glycolytic pathways (Ramseier, 1996; Sarkar et al., 2008; Ramseier et al., 2010). In a study from Zhu et al., Cra was first engineered for achieving high product concentration through error-prone PCR. After screening and mutation site integration, a high succinic acid producing mutant strain (Cra-R57K/A58G/G59Q/R60Q/S75H/T76Y/D148I/R149I), was obtained. This strain (*E. coli* W1485 (*ΔpflAB, ΔldhA, ΔptsG*)/pTrc-mutant *cra*) produced succinic acid to a concentration of 79.8 g/L, which was 22.8% higher than a control strain (*E. coli* W1485 (*ΔpflAB, ΔldhA, ΔptsG*)/pTrc) (Zhu et al., 2016). Further studies demonstrated that the significant increase of succinic acid in a Cra mutant may be caused by the activation of PEP carboxylation, reductive branches of the TCA cycle and the glyoxylate pathway (Zhu et al., 2016). Meanwhile, Li et al. first reported a positive correlation between succinic acid production and the affinity of Cra for its effector fructose-1,6-bisphosphate (FBP) in *E. coli* (Wei et al., 2016). To heighten this affinity, a semi-rational strategy based on computer-assisted virtual saturation mutagenesis was designed, and a Cra mutant (D101R/D148R/G274R) was obtained. Compared with wild-type Cra (the *K*_*d*_ of Cra-FBP was 1,360 ± 6.2 nM), the triple mutant Cra showed a high affinity with its effector fructose-1,6-bisphosphate (FBP) (the *K*_*d*_ of Cra-FBP was 154.2 ± 4.3 nM). Further experimental results indicated that this enhanced affinity increased the activation of succinic acid biosynthetic pathways, especially *pck* and *aceB*, and led to a succinic acid concentration of 92.7 g/L in engineered strain (*E. coli* W1485 (*ΔpflAB, ΔldhA, ΔptsG*)/pTrc99A-*cra* D101R/D148R/G274R), which was 34% higher than a control strain (*E. coli* W1485 (*ΔpflAB, ΔldhA, ΔptsG*)/pTrc99A) (Wei et al., 2016). The advantage of using global transcription factors is that they can regulate the expression of multiple pathway-related genes (Wu et al., 2007; Wei et al., 2016). In conclusion, these results demonstrate the great potentials of global transcription factors combined with the traditional metabolic engineering strategies in succinic acid production process, and this strategy can also be used as a reference for other valuable chemical biosynthetic pathways.

### 3.2 Balancing the Redox Ratio (NADH/NAD^+^)

The intracellular redox ratio is closely related to metabolite profiles, membrane transport, microbial fitness and cellular functions (Berríos-Rivera et al., 2004; Singh et al., 2009). The redox ratio is mainly reflected by the intracellular NADH/NAD^+^ ratio, which is influenced by several factors, such as the physiological state of a given strain, the oxidation state of carbon sources, and the expression levels of NADH/NAD^+^-related enzymes (such as formate dehydrogenase, membrane-bound transhydrogenase, NAD^+^ dehydrogenase, terminal oxidases and lactic dehydrogenase) (Lin et al., 2005a; Singh et al., 2009). In microbes, both NADH and NAD^+^ are kept in balance. NADH is oxidized to NAD^+^ through the oxidative phosphorylation process or fermentation reactions. NADH provides the reducing power for reductive product formation and NAD^+^ serves as an electron acceptor during substrate degradation (Li et al., 2017). In addition, the appropriate NADH/NAD^+^ ratio is a major prerequisite of many biosynthetic processes, and it is also crucial for succinic acid production (Zhang Y. et al., 2009; Li et al., 2017).

Taking *E. coli* as an example, lactate dehydrogenase (encoded by *ldhA*), which drives the formation of lactate, and pyruvate formate lyase (encoded by *pflB*), which drives the formation of formate and acetyl-CoA derivants (acetate, ethanol), were deleted to redirect the carbon flux towards succinic acid under anaerobic conditions (Singh et al., 2010). This mutant was termed *E. coli* NZN111, and it has been considered an excellent candidate as a succinic acid producer (Hui et al., 2009). However, *E. coli* NZN111 lost its ability to ferment glucose anaerobically and accumulates high levels of pyruvate and NADH intracellularly (Wu et al., 2007; Ma et al., 2013). It has been reported that *E. coli* NZN111 growth was impaired mainly due to the anomalously high NADH/NAD^+^ ratio *in vivo*, which was shown to be three times higher than a wild-type *E. coli* strain (Singh et al., 2009; Singh et al., 2010). To reduce this NADH/NAD^+^ ratio, an effective genomic library-based screening approach was employed, and genes including *pfkB* (encoding phosphofructokinase II), *marA* (encoding DNA-binding transcriptional repressor), and *moaE* (encoding the subunit of molybdopterin synthase), were shown to be significant for balancing the redox ratio. Overexpression of these genes was beneficial for succinic acid production, especially *pfkB*, and led to a 7.8-fold increase in M9+10 g/L glucose medium under microaerobic conditions (that means controlled dosing of small amount of air or oxygen into reactor).

Conversely, deficiency in NADH has a negative impact on succinic acid biosynthesis as well. Except for the glyoxylate shunt pathway, conversion of phosphoenolpyruvate, pyruvate or acetyl-CoA to one molecule of succinic acid requires a minimum of two molecules of reducing equivalents (Lin et al., 2005c). However, only two molecules of NADH can be obtained from one molecule of glucose through the glycolytic pathway. Thus, the shortage of NADH limits the succinic acid production to a theoretical yield of 1 mol/mol glucose (Zhu et al., 2014). One solution is to use the glyoxylate shunt pathway (mentioned in section 2), which has a theoretical yield of 1.7 mol/mol glucose (Vemuri et al., 2002). Another solution is to provide more NADH. NAD^+^-dependent formate dehydrogenase can convert one molecule of formate into one molecule of CO_2_ and NADH. In this reaction, formate is one of the main by-products produced by succinic acid producers, while CO_2_ and NADH are necessary for succinic acid synthesis. When using glucose as a carbon source, the expression of formate dehydrogenase can double the maximum yield of NADH, from 2 to 4 mol NADH/mol glucose consumed (Berríos-Rivera et al., 2004). In Grant et al., *fdh* (encoding NAD^+^-dependent formate dehydrogenase) from *C. boidiniiwas* and *pycA* (encoding pyruvate carboxylase) from *L. lactis* were co-expressed in *E. coli* MG1655 (*ΔadhE, ΔldhA, ΔiclR, Δack-pta*) under the control of the same promoters (Thakker et al., 2011). Compared with the overexpression of *pyc* alone, this co-expression strain had better succinic acid production capacity. The succinic acid yield at 24 h showed a 1.9-fold increase, from 176 to 334 mM, and the succinic acid productivity showed a 2-fold increase, from 1 to 2 g/L/h. Meanwhile the byproduct formate concentration showed a significant decrease, from 17 mM to 0–3 mM. This indicated that higher NADH availability conditions significantly changed the final metabolite concentration pattern and promoted an increase in the contents of succinic acid. Additionally, providing carbon sources with different oxidation states is also an optional method. Sorbitol, for instance, has a higher NADH maximum theoretical yield (3 mol NADH/mol sorbitol) than glucose (Berríos-Rivera et al., 2004). It has been reported that using sorbitol can also generate higher yield and productivity of succinic acid (Chatterjee et al., 2001).

### 3.3 Optimizing CO_2_ Supplementation

In succinic acid biosynthesis, HCO_3_^-^ is the major carboxylation substrate. During fermentation processes, CO_2_ that is dissolved in the liquid medium, first crosses the cell membrane through passive diffusion, and is then converted into HCO_3_^-^ in the cytoplasm to participate in carboxylation (Lu et al., 2009). However, the low concentration of CO_2_ in the growth medium is the critical limiting step in succinic acid production (Zhu et al., 2015). To date, several strategies have been attempted to improve succinic acid production by optimizing CO_2_ supplementation and several satisfying results have been achieved.

#### 3.3.1 Promoting the Intracellular Conversion of HCO_3_^-^ and CO_2_

Under anaerobic fermentation conditions, both CO_2_ or HCO_3_^-^ can serve as a carboxylation substrate for PEP carboxykinase (PCK), but PCK prefers to use CO_2_, and its catalytic velocity with CO_2_ was shown to be 7.6-fold higher than that with HCO_3_^-^ (Cotelesage et al., 2007). Therefore, promoting the intracellular interconversion of HCO_3_^-^ and CO_2_ is a way to improve its catalytic efficiency. Carbonic anhydrase (CA), which is an important component of the carboxysome, efficiently converts HCO_3_^-^ to CO_2_ (Cotelesage et al., 2007). For improving the carboxylation velocity of PCK, Xiao et al. introduced the CA gene from *Synechococcus* sp. PCC7002 into *E. coli* Suc-T110 *Δppc.* Due to the increase of the local CO_2_ concentration, the carboxylation rate of PCK showed a 1.6-fold increase from 2.46 to 3.92 μmol/min/mg protein. As a result of CA gene overexpression, the succinic acid titer at 36 h showed a 35% increase as well (Xiao et al., 2017). These results demonstrated that carbonic anhydrase was useful for improving succinic acid productivity.

#### 3.3.2 Enhancing HCO_3_^-^ Transmembrane Transport

As the major carboxylation substrate, HCO_3_^-^ can also cross the cell membrane through passive diffusion, but the rate is very slow, limiting its direct utilization (Badger and Price, 2003). One feasible strategy to increase intracellular HCO_3_^-^ concentration is to use bicarbonate transporters. Among these, SbtA and BicA have been shown to be more efficient (Price et al., 2004). BicA, which was identified from *Synechococcus* sp. PCC7002, is a Na^+^-dependent transporter, and it has low transport affinity but a high flux rate (Price et al., 2004). SbtA-mediated transport, which was identified from *Synechocystis* PCC6803, is also Na^+^-dependent. Meanwhile, it can be induced by low CO_2_ levels and shows a relatively high affinity for HCO_3_^-^ (Shibata et al., 2002; Zhu et al., 2015). To optimize succinic acid production, Xiao et al. overexpressed the *bicA* and *pck* genes in *E. coli*, and reported that the final cell density and succinic acid titers at 36 h were 29 and 22% higher than those of control strains, respectively (Xiao et al., 2017). Zhu et al. contrasted SbtA and BicA in *E. coli* AFP111, and demonstrated that overexpression of *sbtA* or/and *bicA* genes showed a positive effect on CO_2_ absorption but a negative effect on succinic acid biosynthesis. Meanwhile, they found that the succinic acid concentration was improved only when bicarbonate transporters and carboxylase (PCK or PPC) were co-expressed. Among these, co-overexpression of the *sbtA* and *pck* genes was superior, and led to a 15% increase in succinic production (Zhu et al., 2015). For further optimization, Yu et al. balanced HCO_3_^-^ transport and fixation to maximize succinic acid titer. They demonstrated that the highest succinic acid production was obtained when the *sbtA*, *bicA*, and *ppc* genes were co-expressed under the control of the weak P4 promoter and the *pck* gene under the control of the strong P19 promoter. This led to a 1.4-fold increase in succinic acid production from 65 to 89.4 g/L, and a 1.3-fold increase in succinic acid yield from 0.98 to 1.27 mol/mol glucose (Yu et al., 2016). All of these results indicated the necessity of CO_2_ supplementation in succinic acid biosynthesis.

## 4 Conclusion and Perspectives

Succinic acid is an important four-carbon building-block chemical that can be used as the precursor of numerous products, including biodegradable plastics, feed additives, green solvents, and pharmaceutical products. As an alternative to environmentally unfriendly traditional method, biosynthesis is a promising means for succinic acid production and has become increasingly mature as well. This review summarized different succinic acid biosynthesis pathways, key enzymes, and the metabolic engineering approaches, particularly those developed for adjusting the carbon flux, balancing the redox ratio, and CO_2_ supplementation for succinic acid production. It needs to be clear that one or more strategies have been applied jointly to obtain high performance strains in practical application. To date, some efficient succinic acid producers have been obtained and applied in industry, but several problems still exist, such as the low robustness of engineered bacteria or the high cost of downstream product recovery. Thus, increasing attention should be paid to the following prospects in future research.

Firstly, an economical fermentation process should be further explored. Most succinic acid producing strains prefer neutral pH conditions, but succinic acid formation acidifies the medium. To keep a neutral pH, a common method is the conversion of succinic acid into succinate by titration with bases during fermentation. However, generation of salts increases the difficulty and cost of post-processing for succinic acid production. Compared with feedstock costs and some other upstream costs, the downstream costs are also considerable, and represent about 30–40% of the total production costs (López-Garzón and Straathof, 2014). One solution is to develop low pH (below or close to the p*K*_a_) fermentation strategies, which can minimize the consumption of bases, reduce the generation of salts and avoid re-conversion of succinate into succinic acid. However, the highest succinic acid concentration has so far been achieved at neutral pH, not at low pH. The main reason is the low tolerance to low pH of most succinic acid producers, such as *C. glutamicum*, *E. coli*, *M. succiniciproducens*, *B. succiniciproducens* and *A. succinogenes* (Ahn et al., 2016). Therefore, to reduce the overall production costs, development of a competitive acid-tolerant strain is critical for enabling bio-based production of succinic acid. Meanwhile, more attention should be paid to revealing acid-tolerant mechanisms, which are meaningful for the production of any carboxylic acid.

Furthermore, efficient biosynthesis process should be further explored. Making a detailed analyze and comparison of the seven succinic acid biosynthetic pathways is very important for understanding and designing greater efficiency biosynthesis pathway. And no matter which pathway is selected, the metabolic process for its production is a multigene pathway. Key enzyme overexpression is the traditional genetic engineering strategy used to overcome this, but the enzyme expression level in a multistep pathway is not simply “the more the better”. Liu et al. demonstrated the importance of metabolic balancing in a multistep biosynthetic pathway (Liu et al., 2016). Meanwhile, plasmid-based multigene over-expression systems can also lead to genetic instability and high cellular burdens. Thus, a functional balance between enzymes and the improvement of host stability in succinic acid biosynthetic processes should be considered. Some strategies are valuable, such as chromosomal integration, promoters or RBS engineering, microbial host engineering, and dynamic control of the pathway of interest.
